# Value of strain analysis with feature tracking in dobutamine stress cardiac magnetic resonance imaging for detecting coronary artery disease

**DOI:** 10.1186/1532-429X-16-S1-P179

**Published:** 2014-01-16

**Authors:** Christopher Schneeweis, Jianxing Qiu, Bernhard Schnackenburg, Alexander Berger, Sebastian Kelle, Eckart Fleck, Rolf Gebker

**Affiliations:** 1 Cardiology, German Heart Institute Berlin, Berlin, Germany; 2 Department of Radiology,, Peking University First Hospital, Beijing, China; 3 Philips Research Hamburg, Hamburg, Germany

## Background

Dobutamine stress magnetic resonance (DSMR) has been established for the detection of coronary artery disease (CAD) due to its high diagnostic accuracy and prognostic value. The novel technique feature tracking (FT) analyses regional myocardial strain and thereby offers detailed information about myocardial deformation. The purpose of this study was to examine how FT based circumferential strain (CS) compares to visually detected wall motion abnormalities (WMA) during DSMR and whether it adds beneficial information for detecting CAD.

## Methods

A total of 25 patients (72% male; mean age 64 ± 8) with suspected or known CAD underwent a standardized high-dose DSMR protocol at 1.5 T. CMR images were acquired at rest and during stress in three short-axis views (SAX; apical, medial, basal). None of the patients had evidence for WMAs or impaired left ventricular function at rest or scar tissue based on late gadolinium enhancement (LGE). For strain analysis the SAX planes were divided into 16 segments (n = 400 segments). All patients underwent x-ray coronary angiography for clinical reasons. Patients without WMA during DSMR and exclusion of CAD by coronary angiography were defined as normal and served as control (10 patients, 160 segments). 15 patients developed WMA during DSMR (34 segments). Additionally, based on angiographic findings, segments that were supplied by a vessel with >75% were defined as stenotic (64 segments). The remaining segments, which did not develop a WMA or were supplied by a vessel <50% narrowing, were considered as remote segments (161 segments).

## Results

**Figure 1:**
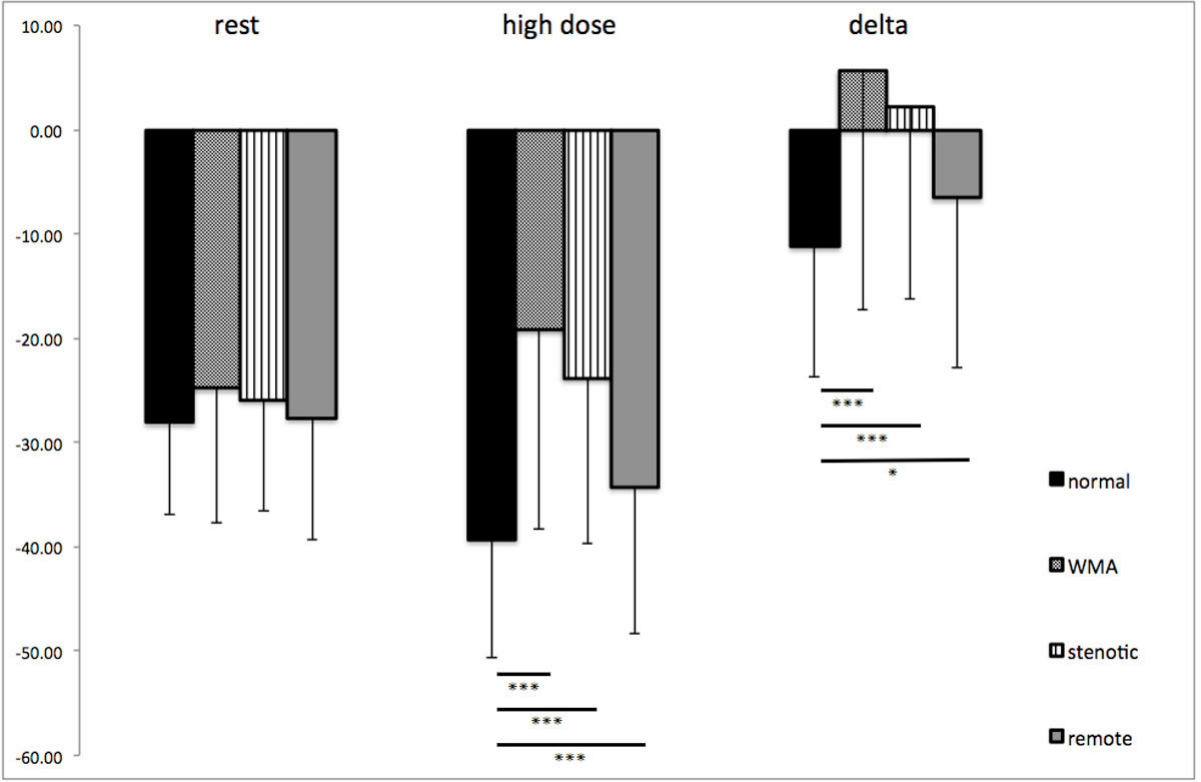


**Figure 2:**
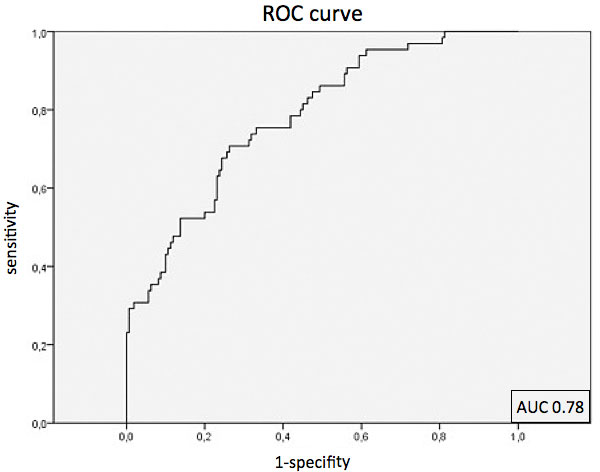


At rest no differences in CS were observed between groups (normal: -28.1 ± 8.9; WMA: -24.8 ± 12.9; stenotic: 25.8 ± 10.6; remote: 27.7 ± 11.7%; p = 0.23). High dose dobutamine stress revealed highly significant differences between normal segments and segments with WMA (normal: -39.3 ± 11.3%; WMA: 19.2 ± 19.1%; p < 0.001) as well as between normal and stenotic and normal and remote segments (stenotic: -23.8 ± 15.9%, p < 0.001; remote: -34.2 ± 14.1%, p < 0.001). Additionally, analysis of the absolute change in CS between rest and stress showed significant differences for normal segments vs. segments with WMA (Δ normal: -11.2 ± 12.6%, Δ WMA: 5.6 ± 22.9%; p = 0.001), as well as between normal and stenotic segments (Δ stenotic: 1.9 ± 18.4%; p < 0.001) and between normal and remote segments (Δ remote: -6.6 ± 16.3%; p = 0.027). CS results are summarized in Figure 1. ROC analysis of CS during maximum DSMR differentiated normal from stenotic segments with a sensitivity of 75% and specificity of 67% using a cutoff -33.2% with an area under the curve of 0.78 (Figure 2).

## Conclusions

FT based CS analysis demonstrated significant differences between normal segments and segments with WMA in DSMR. Additionally, FT detected significantly impaired CS in stenotic segments compared to normal segments. FT based CS may improve the diagnostic accuracy of DSMR for detection of ischemia.

## Funding

Nu funding.

